# Growth, structure, morphology, and magnetic properties of Ni ferrite films

**DOI:** 10.1186/1556-276X-8-196

**Published:** 2013-04-27

**Authors:** Chunhui Dong, Gaoxue Wang, Dangwei Guo, Changjun Jiang, Desheng Xue

**Affiliations:** 1 Key Lab for Magnetism and Magnetic Materials of the Ministry of Education, Lanzhou University, Lanzhou 730000, People’s Republic of China

**Keywords:** Crystal growth, Sputtering, Thin films, NiFe_2_O_4_, Spinel structure, 75.70.-i, 75.70.Ak, 75.60.Ej

## Abstract

The morphology, structure, and magnetic properties of nickel ferrite (NiFe_2_O_4_) films fabricated by radio frequency magnetron sputtering on Si(111) substrate have been investigated as functions of film thickness. Prepared films that have not undergone post-annealing show the better spinel crystal structure with increasing growth time. Meanwhile, the size of grain also increases, which induces the change of magnetic properties: saturation magnetization increased and coercivity increased at first and then decreased. Note that the sample of 10-nm thickness is the superparamagnetic property. Transmission electron microscopy displays that the film grew with a disorder structure at initial growth, then forms spinel crystal structure as its thickness increases, which is relative to lattice matching between substrate Si and NiFe_2_O_4_.

## Background

Ferrite films have been widely used in computer memory chips, magnetic recording media, frequency filters, and many branches of telecommunication and electronic engineering. In particular, Ni ferrite (NiFe_2_O_4_) films with spinel structure were currently of great interest due to their high magnetic permeability, high resistivity, and low losses, making itself a promising material for high-frequency applications. Many methods have been carried out to fabricate ferrites, such as molecular beam epitaxy [1], pulsed laser deposition [2,3], spin-spray [4,5], sol–gel [6], electrochemical deposition [7], direct liquid phase precipitation [8], hydrothermal growth [9,10], and sputtering [11,12]. Researches on structural and magnetic properties of ferrites have been devoted recently. Li et al. [11] have reported that NiZn ferrite can be fabricated under low temperature. However, the magnetic properties of NiZn ferrite films fabricated under low temperature were not as good as bulk status, usually amorphous or with high coercivity (*H*_c_) and low saturation magnetization (*M*_s_) [11]. Usually, high-temperature post-heating treatments or *in-situ* heating was needed to obtain a better spinel structure and soft magnetic property [11]. But heating treatment was detrimental to the electric circuit integrations, which limited the applications of ferrite films as promising materials for high-frequency devices. Therefore, it was significant to investigate the effect of growth at room temperature (RT) on the structure and magnetic properties of ferrite films.

In this work, Ni ferrite films with different thicknesses (10, 50, 100, 500, and 1,000 nm) were fabricated under RT. Structure and magnetic properties were investigated as functions of thickness. Note that the 10-nm film showed superparamagnetism, different from the other samples (ferromagnetism), which was believed to be caused by the disordered layer discovered by transmission electron microscopy (TEM).

## Methods

NiFe_2_O_4_ ferrite films were deposited onto 20 mm × 20 mm Si(111) substrates attached to a water-cooling system by radio frequency magnetron sputtering with a base pressure below 5 × 10^-5^ Pa. The mixed gas of argon and oxygen was used as the sputtering gas at total pressure of 2.5 Pa. The sample thickness was controlled by deposition duration. The crystal structure was checked by X-ray diffraction (XRD; X’Pert PRO PHILIPS (Almelo, Netherlands) with CuK*α* radiation). The images of the surface microstructure were taken using a field emission scanning electron microscope (SEM; S-4800, Hitachi, Ltd., Tokyo, Japan). The magnetic properties were measured using the MPMS magnetometer based on a superconducting quantum interference device (SQUID). The micrograph of the cross-section of the 500-nm NiFe_2_O_4_ film was taken by TEM (Tecnai TMG2F30, FEI, Hillsboro, OR, USA).

## Results and discussion

**Figure 1:**
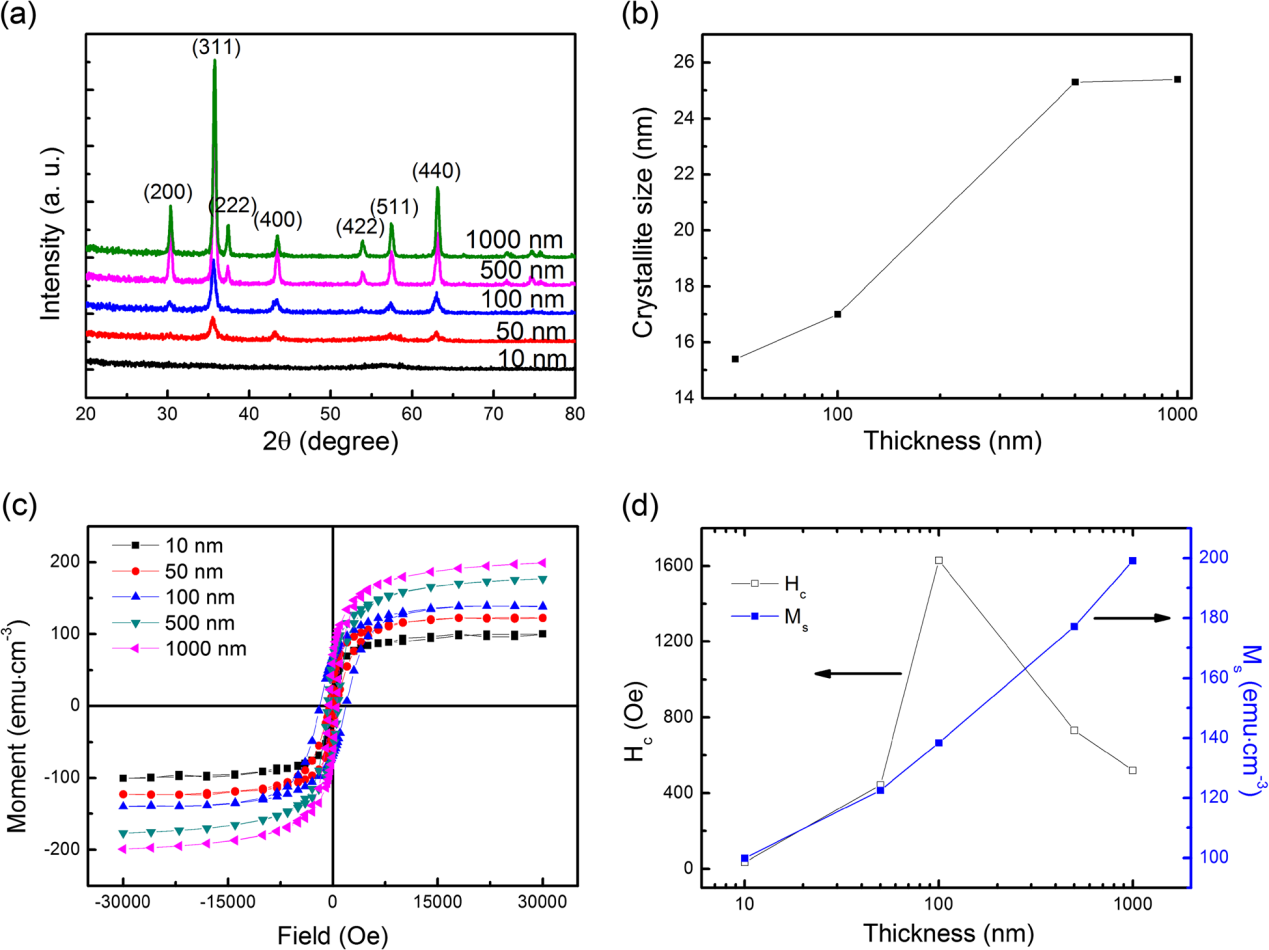
**Ferrite films with different thicknesses of 10, 50, 100, 500, and 1,000 nm. **XRD patterns (**a**), crystallite sizes (**b**), and hysteresis loops (**c**). Thickness dependence of *M*_s _and *H*_c _of the NiFe_2_O_4 _films at RT (**d**).

XRD analysis was performed at RT after the films were fabricated. No annealing procedure was carried out. Figure 1a shows the XRD patterns of the prepared ferrite films. Films thicker than 50 nm are well crystallized with the spinel crystal structure (JCPDS card no. 54–0964). No secondary phase was detected, which indicates that the films are pure spinel nickel ferrite. No obvious diffraction peak was observed in the 10-nm film, suggesting an amorphous-like state. Figure 1b shows the crystallite sizes calculated by Debye-Scherrer formula [13]. Crystallite size increases rapidly from 15 nm in 50-nm film to 25 nm in 500-nm one. When the film thickness exceeded 500 nm, the crystallite size remains almost unchanged, indicating that crystal growth is in equilibrium status.

Figure 1c shows the in-plane hysteresis loops of the films at different thicknesses at RT. The *H*_c_ and *M*_s_ with various Ni ferrite film thicknesses are summarized in Figure 1d. *M*_s_ increases monotonically with increasing ferrite film thickness, while *H*_c_ increases sharply with the film thickness less than 100 nm and then decreases hugely at 500 nm. Note that the 10-nm film shows superparamagnetic behavior with almost zero *H*_c_[14].

Generally speaking, the *M*_s_ of ferrite is related to its crystal structure. For spinel ferrite films, ferromagnetism is induced by oxygen superexchange effect between sites A and B [15]. Therefore, the better spinel crystal structure is, the larger *M*_s_ is. In our work, according to the XRD results, the crystal structure becomes better with increasing film thickness, which results in the increase of *M*_s_. However, *H*_c_ is attributed to many factors such as grain size, the magnetization (*M*) reversal process, etc.

**Figure 2:**
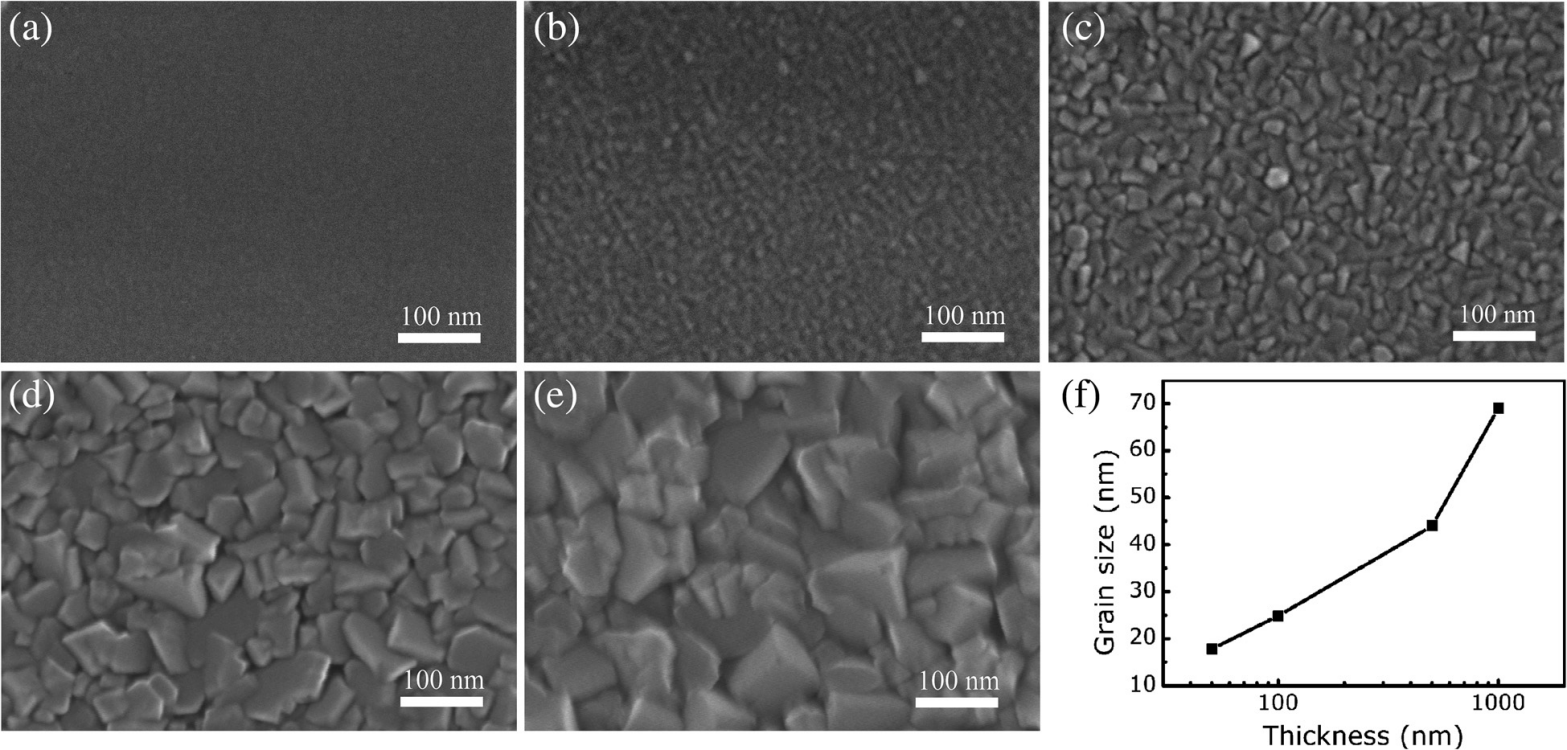
**SEM images of ferrite films with different thicknesses. **10 (**a**), 50 (**b**), 100 (**c**), 500 (**d**), and 1,000 nm (**e**). Thickness dependence of grain size (**f**).

In order to understand the change of *H*_c_ further, the microstructures of the ferrite films were investigated using SEM. The surface images of the films with different thicknesses are shown in Figure 2. It is obvious that film thickness affects grain size hugely, which increases with increase in thickness. *H*_c_ is related to the reversal mechanism of *M*. Broadly speaking, *M* reversal mechanism varies with grain size. When grain size is smaller than the single-domain critical size, *M* reversal mechanism can be described as coherent rotation. Due to this mechanism, *H*_c_ increases with increasing grain size [16]. When the grain size is much bigger than single-domain critical size, *M* reversal mechanism turns into a domain wall motion; therefore, *H*_c_ decreases as grain size increases [12]. Moreover, the grain boundary volume decreases due to the increase of grain size. Therefore, the ‘pinning’ effect of domain wall among the grains’ boundary is weakened when thickness increases, which makes the *M* reverse easier and causes *H*_c_ to decrease [11]. Therefore, the *H*_c_ firstly increases when thickness is less than 100 nm, then decreases with the increasing thickness, which results from the competition between the above factors.

**Figure 3:**
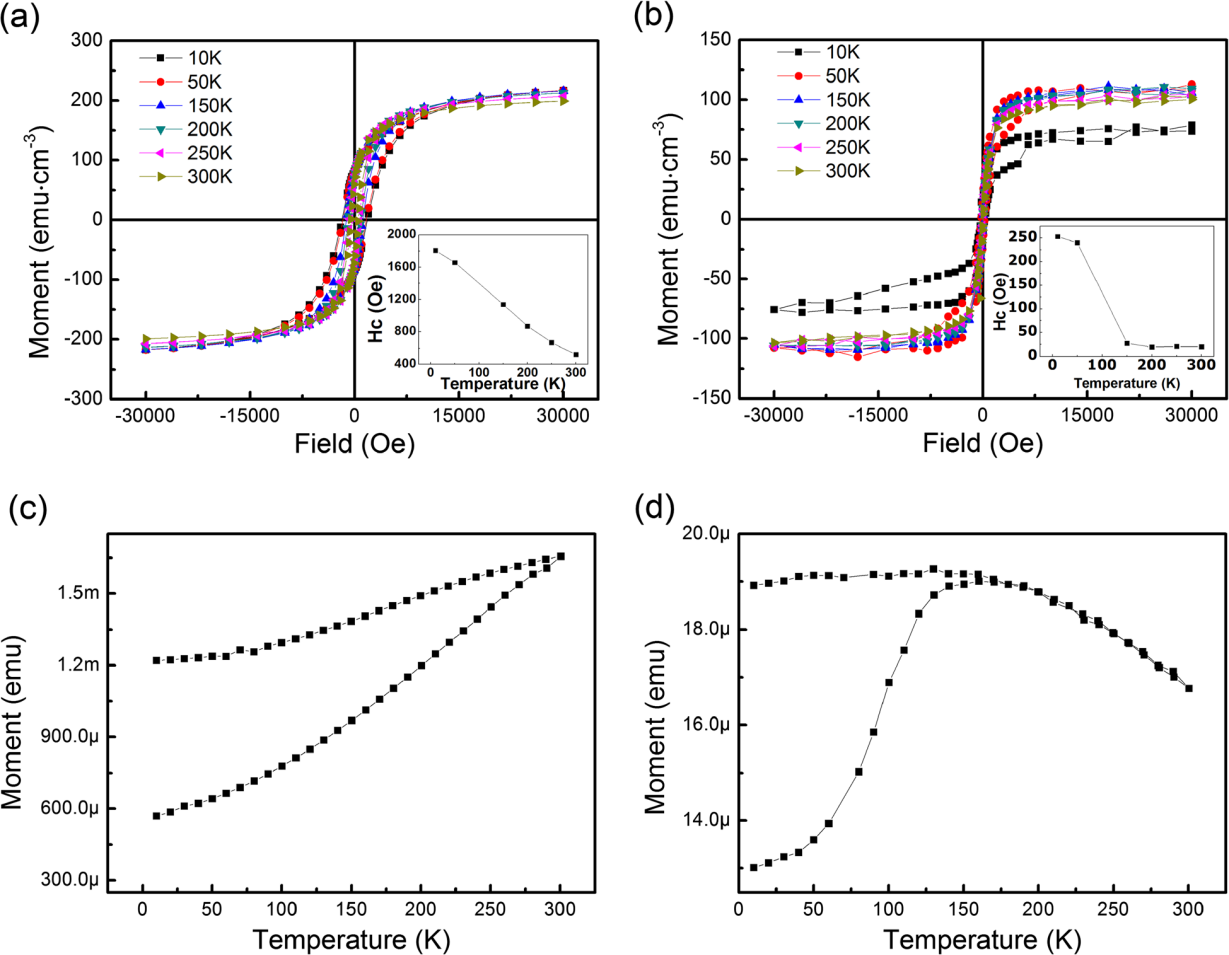
**Hysteresis loops of the films in 1,000 (a) and 10 nm (b) under different temperatures. **ZFC (lower branch) and FC (upper branch) *M *as a function of temperature measured on samples of 1,000 (**c**) and 10 nm (**d**).

In order to investigate the effect of growth on the magnetic properties further, in-plane hysteresis loops and zero-field-cooling (ZFC)-field-cooling (FC) curves of 1,000- and 10-nm films were measured. Figure 3a,b shows the hysteresis loops under different temperatures. The *H*_c_ dependence of temperature summarized in the insets reveals different trends. For the 10-nm film, *H*_c_ decreases sharply from 230 Oe at 50 K to almost 0 Oe at 150 K, while the *H*_c_ of 1,000-nm film decreases monotonically with increasing temperature. This can be explained by the FC-ZFC curves shown in Figure 3c,d. The *M*_ZFC_ was measured on warming from 10 to 300 K, whereas *M*_FC_ was recorded during the subsequent cooling. The applied field during the measurement was constantly 1,000 Oe. For the 1,000-nm film, no blocking temperature (*T*_B_) was found, indicating the typical ferromagnetic property [14], while *T*_B_ at 170 K is observed in the 10-nm film. Below *T*_B_, the film shows ferromagnetic behavior, where the thermal energy is insufficient to compete the energy of turning magnetic moments to external magnetic field direction. However, when the temperature rises to 170 K, thermal energy is high enough to induce unfixed direction of magnetic moments. Therefore, *H*_c_ is almost zero [3,14].

**Figure 4:**
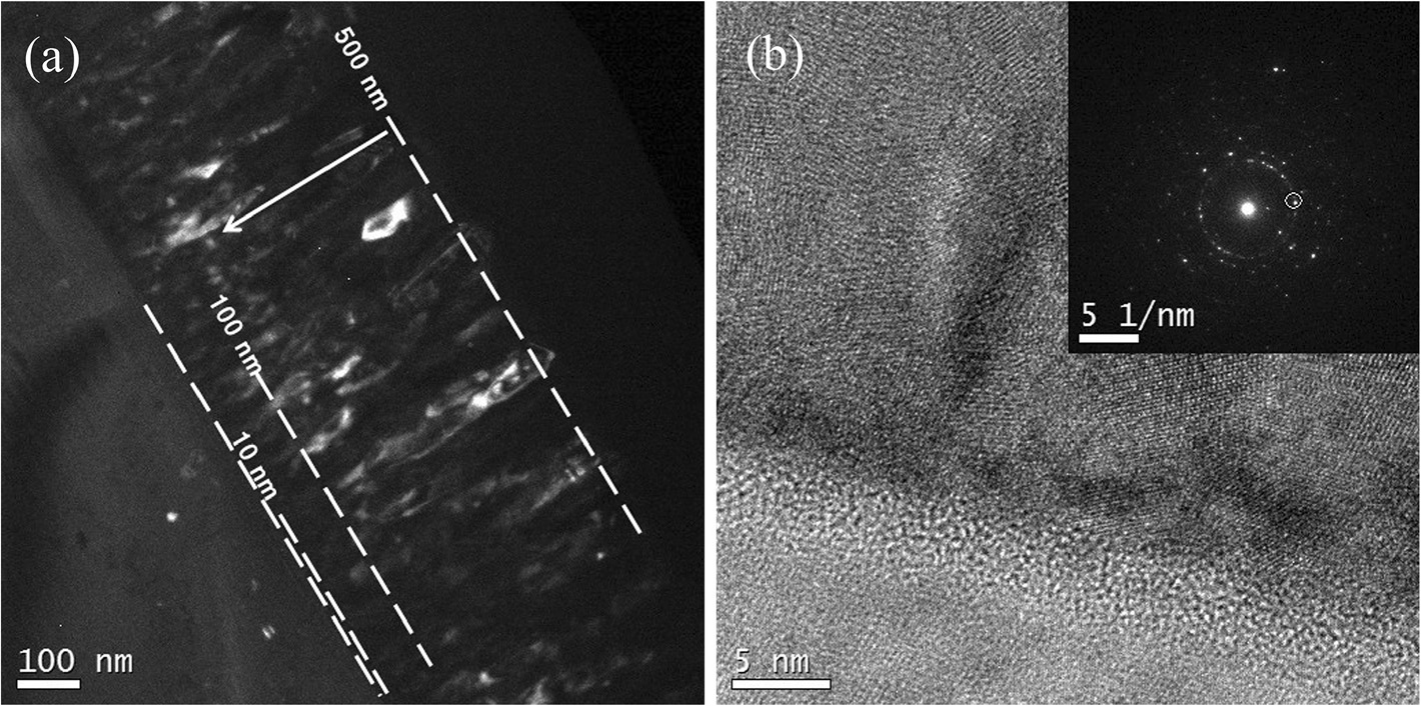
**TEM images of the 500-nm ferrite film. **Dark-field cross-section image (**a**) and the HRTEM image (**b**).

In order to understand the effect of film growth on structure and magnetic properties, a micrograph of the cross-section of 500-nm NiFe_2_O_4_ film was taken by TEM. Figure 4a is the dark-field cross-section image. Though the crystal structure of the 500-nm Ni ferrite shows good spinel phase, the TEM image reveals a different microstructure as the thickness of film increases. In the 10-nm film, the crystalline is hardly found; while for the film thickness of 100 nm, crystallites are observed obviously, and the crystallite size increases when thickness increased. Figure 4b shows the high-resolution transmission electron microscopic (HRTEM) image. A disorder layer at the bottom of the ferrite layer has been found. Due to the big mismatch between the lattice constants of NiFe_2_O_4_ (8.337 Å) and Si (5.431 Å), the crystal orientation is disorganized [3]. With the development of the growth process, mass islands of crystallite form, and then the islands gradually merged together into big ones. Finally three-dimensional crystals fill the space available and form the dense columnar structure [3,17]. TEM result also agrees with the results of XRD and SQUID. The *M*_s_ of the ferrite films increases with the increase of the crystallite size [11,12]. When the film thickness is less than 10 nm, thermal energy interrupts the magnetic moment orientation due to small grain size, which shows superparamagnetic effect. With increasing film thickness, spinel structure is formed and crystallite size increases, which results in the decrease in the full width at half maximum of the X-ray spectral peaks and the increase of *M*_s_.

## Conclusions

Ni ferrite films with different thicknesses were fabricated under RT. Structure and magnetic properties of Ni ferrite films were investigated as functions of thickness: the 10-nm film exhibits superparamagnetism; *M*_s_ increases monotonically, while *H*_c_ first increases then decreases as the film thickness increases. The SEM and TEM images were taken to investigate the underlying magnetic mechanism. A disordered layer at the bottom of the ferrite layer can be seen in the TEM image; this layer may probably be responsible for the superparamagnetic behavior of the 10-nm film.

## Competing interests

The authors declare that they have no competing interests.

## Authors’ contributions

CD fabricated the NiFe_2_O_4_ films, performed the measurements, and wrote the manuscript. CJ analyzed the results and wrote the manuscript. GW and DG helped grow and measure the films. DX supervised the overall study. All authors read and approved the final manuscript.
